# Con-FLIC: concurrent measurement of feeding behaviors and food consumption in *Drosophila* at single-fly resolution

**DOI:** 10.17912/micropub.biology.001128

**Published:** 2024-02-28

**Authors:** Mubaraq Opoola, Lucas Fitzgerald, Dae-Sung Hwangbo

**Affiliations:** 1 Department of Biology, University of Louisville, Louisville, Kentucky, United States

## Abstract

Accurate quantification of food intake and feeding behaviors are essential for understanding various biological processes, such as metabolism and aging. Currently, no methods allow for the concurrent measurement of both parameters for the same individual flies. Here, we couple Con-Ex ( Con sumption- Ex cretion) and FLIC ( F ly L iquid-Food I nteraction C ounter), previously developed to measure food consumption and various feeding behaviors, respectively, into a single platform that we named Con-FLIC. Using starvation as a known condition that changes food intake and feeding behaviors, we validate that Con-FLIC enables concurrent measurement of feeding behaviors and food intake in *Drosophila* at a single-fly resolution. We expect that Con-FLIC will be an easy non-invasive option to quantify food consumption and feeding behaviors concurrently in the same individual flies.

**Figure 1. Concurrent quantification of food consumption and feeding behaviors using Con-FLIC:**
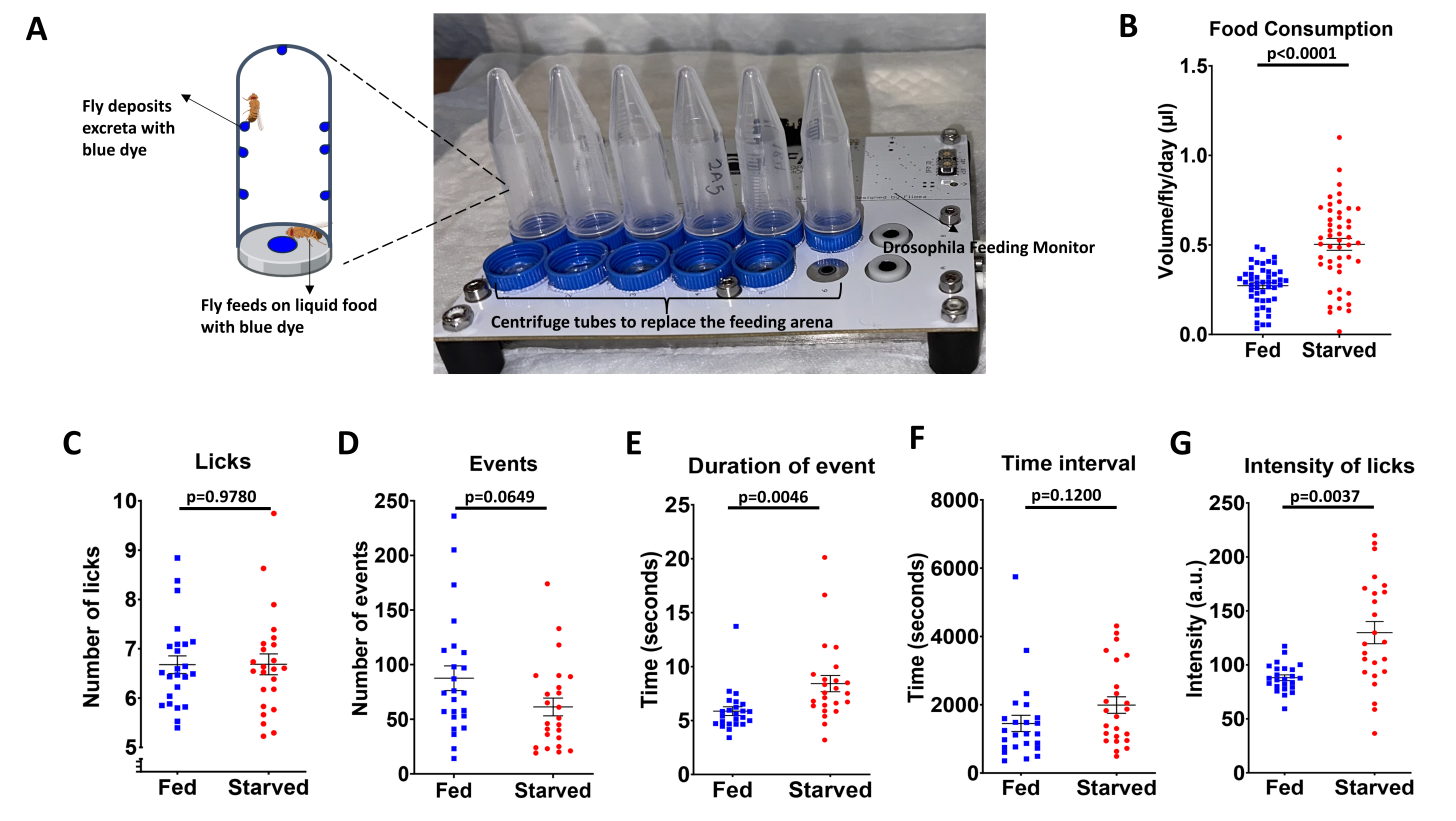
( **A** ) Illustration of the Con-FLIC setup. Only DFM part of the FLIC is shown in the figure. Single-housed flies feed on liquid food with 1% blue dye (FD&C Blue #1) in the FLIC component of Con-FLIC and deposit blue-dyed excreta on the walls of the centrifuge tubes in the Con-Ex component of Con-FLIC. ( **B** ) Food consumption quantification obtained from the Con-Ex component of Con-FLIC. ( **C-G** ) Feeding behavior quantification obtained from the FLIC component of Con-FLIC. For both measurements, male flies (~3 days old, *w ^1118^* ) that had either fed on 20% sucrose food (n=24) or were starved on agar (n=24) for 24 hours were transferred to the Con-FLIC machine without CO _2_ anesthesia and allowed to consume 20% sucrose food mixed with 1% blue dye (FD&C Blue #1) for another 24 hours. After 24 hours, flies’ excreta were harvested from the Con-Ex component of Con-FLIC for food quantification, and the feeding behavior data were collected from the FLIC component of Con-FLIC for feeding behavior analysis. The same set of flies were used to generate the data in all the figures. Significance (p-value) was determined using unpaired t-test for food consumption and feeding behaviors. Error bars indicate the standard error of the mean (SEM).

## Description

Feeding is an essential behavior for organisms to survive and regulate diverse biological processes, including energy balance, locomotion, aging, and fecundity. Therefore, accurate quantification of food intake and feeding-associated behavioral parameters, such as feeding bout numbers and their duration, is crucial for understanding the impact of food and diet on these biological processes (Bonfini et al., 2021; Carvalho et al., 2005; Deshpande et al., 2014; Diegelmann et al., 2017; Ja et al., 2007) .

In *Drosophila* , since the amount of food intake in individual flies is at a micro-scale, making it challenging to measure directly, various tools have been developed to quantify the amount indirectly. The Con-Ex assay quantifies the food intake of flies by measuring the dye excreted following the ingestion of dye-labeled food (Shell & Grotewiel, 2022; Shell et al., 2021; Shell et al., 2018) . Another system called FLIC quantifies various feeding-associated behavioral parameters, such as the number of flies’ licks to food and its duration, by detecting electronic signals generated when flies contact the food, mostly with their proboscis. These behavioral parameters may provide insights into the frequency of food intake and meal size, allowing for the extrapolation of food consumption. However, quantification of food consumption inferred from proboscis extension-dependent feeding behaviors using FLIC may be complicated because proboscis extension to food does not always translate to feeding (Itskov et al., 2014; Qi et al., 2015; Yapici et al., 2016) . While the Con-Ex assay is an end-point food intake measurement limited to solid food and cannot assess any feeding-related behaviors, the FLIC system continuously measures feeding behaviors exclusively for liquid food (Ro et al., 2014) . Therefore, although Con-Ex and FLIC can complement each other in measuring food intake alongside feeding behaviors in flies, making a direct comparison poses challenges due to the use of different flies for each measurement, temporal gaps even when the same flies were used, and variations in food types (solid vs. liquid).

Here, we report a new system named Con-FLIC, which combines Con-Ex and FLIC into a single platform. Leveraging the advantages of the Con-Ex assay and the FLIC settings, Con-FLIC is designed to concurrently quantify both feeding behaviors and food consumption for the same individual flies. Briefly, while the FLIC component of the Con-FLIC records data for feeding behaviors, the Con-Ex component of the Con-FLIC collects excretion of flies that consume liquid food supplanted with 1% blue dye (FD&C Blue #1). For the Con-Ex component of the Con-FLIC, the fly arena of the original FLIC is replaced with a 5 mL conical screw tubes (See Methods and Figure 1A for a detailed description of the Con -FLIC configuration).

To validate Con-FLIC, we employed starvation as a known condition that significantly alters both food consumption and feeding behaviors. As a homeostatic response, flies consume more food after starvation (Shell & Grotewiel, 2022; Shell et al., 2021; Shell et al., 2018) . Similarly, flies exhibit an extended contact time with food during the refeeding period after starvation (Itskov et al., 2014; Ro et al., 2014) . To test this in Con-FLIC, young male flies (~3 days old) were first starved in agar-only vials, while another group of flies was fed a 20% sucrose diet. After 24 hours, both groups of flies were individually transferred to the Con-FLIC arena ( Figure 1A ) without CO _2_ anesthesia and allowed to feed on a liquid diet containing 20% sucrose and 1% blue dye for an additional 24 hours. As expected, pre-starved flies significantly increased food consumption during the re-feeding period ( Figure 1B ), validating that Con-FLIC efficiently quantifies the food consumed by individual flies. We then examined five behavioral parameters (licks: the number of feeding licks in the defined range, intensity: signal intensity across all licks, events: the number of groups of contiguous licks in the defined range, duration: the duration of feeding events, time interval: time between feeding events) in the same flies (Ro et al., 2014; Weaver et al., 2023) . Interestingly, neither the number of licks ( Figure 1C, p =0.9780), nor events ( Figure 1D, p =0.0649), as well as the average time interval ( Figure 1F, p =0.1200), showed significant changes after starvation. However, the average duration of events ( Figure 1E, p =0.0046) and the intensity of licks ( Figure 1G, p =0.0037) significantly increased during the refeeding period following the 24 hours of starvation. The increase in the duration of events following starvation is consistent with previous reports (Itskov et al., 2014; Weaver et al., 2023) , suggesting that the modification of the FLIC machine adopted for Con-FLIC does not significantly interfere with flies’ intrinsic feeding behaviors. These data also suggest that the strength or duration (intensity of licks and duration of events) of the fly-food interaction, rather than its frequency, determines the amount of total food consumption, at least under the conditions used in this study.

In this manuscript, we demonstrate the successful integration of two independent tools originally developed for food consumption measurement and feeding-related behaviors into a single unit named Con-FLIC, enabling the concurrent quantification of both traits. As Con-FLIC can measure these traits in the same flies under identical environmental conditions in real-time, it eliminates potential genetic and environmental variables that may serve as confounding factors. While Con-FLIC cannot be used for solid food in its current format, expansion is possible by adopting a similar platform that also utilizes electronic signals but accommodates solid food (Itskov et al., 2014) . We expect that Con-FLIC or its potential variations can serve as an easy, non-invasive tool without sacrificing flies to measure food intake and continuous feeding behaviors in the same individual flies.

## Methods

***Fly Strain and Husbandry***

The *w ^1118^* strain (Bloomington Stock #5905) was used for all experiments. Flies were reared at 25°C under a 12 h:12 h light–dark cycle with 40~60% relative humidity on a standard cornmeal-based medium (Genesee Scientific, Cat#: 66-113) with the addition of 2% (w/v) yeast (Brewer’s yeast, MP Biomedicals).

***Con-FLIC Configuration***

The Con-FLIC combines and modifies features of the FLIC system (Ro et al., 2014) and the Con-Ex method (Shell et al., 2018) . We acquired the commercial version of the FLIC system from Sable Systems International ( https://www.sablesys.com ). Similar to the original FLIC setup, the FLIC component of the Con-FLIC consists of the Drosophila Feeding Monitor (DFM) with a modified fly arena to allow flies to collect excreted dyes from flies, the Master Control Unit (MCU), and the reservoir. The DFM generates raw data on feeding behaviors by quantifying fly-to-food interactions. The MCU collects the raw fly-to-food interaction data from the DFM and processes them for secondary analysis using R. The reservoir supplies liquid food supplemented with blue dye (FD&C Blue #1) at a final concentration of 1% to the DFM. The original fly arena of the FLIC was replaced with a 5 mL centrifuge tube, serving as the hub for the Con-Ex component of the Con-FLIC. A wide hole was carved into the cap of the tube, leaving a small part of the cap behind, just enough to serve as an adhesion surface. Mini-size glue sticks (Elmer’s CraftBond; #E6055) were used to attach the cap to each of the wells on the DFM. This allows for easy screwing of the tube upside down, serving as the housing and the dyed fecal collection apparatus ( Figure 1A ).

***Con-FLIC Assay***

For the starvation pretreatment prior to the Con-FLIC assay, young mated males (~3 days old) were either starved in 1% (w/v) agar-only vials or in 20% (w/v) sucrose vials for 24 hours. Individual flies were then transferred from the vials, without CO _2_ anesthesia, onto the wells of the DFM in the Con-FLIC system, which was supplied with a 20% sucrose solution containing 1% FD&C Blue #1 dye and with 0.005% MgCl _2_ (May et al., 2019) . Con-FLIC assays were conducted inside an incubator at 25°C with a 12:12 hr light:dark (LD) cycle, from 9 AM to 9 PM for the light period and from 9 PM to 9 AM for the dark period. The assay started at 9 AM and ran for 24 hours.

***Con-FLIC Analysis and Visualization***

The raw data collected from the FLIC component of the Con-FLIC were analyzed and visualized

in R (version 4.2.3) and R Studio (version 2023.03.0+386) software using the codes available on the following GitHub ( https://github.com/PletcherLab/FLIC_R_Code ). These codes were employed to calculate various feeding behaviors, including licks, events, duration, time interval, and intensity. The food consumption samples collected from the Con-Ex component of the Con-FLIC were analyzed following the protocol by Shell et al. (2019) with minor modifications.

Briefly, the excreted dye from flies in the tubes was collected by vortexing in 1.5 mL of water, and its concentration was determined by absorbance at 630 nm. Optical density was extrapolated on a standard curve to calculate the volume of food consumed by flies. Figures and statistics (unpaired t-test) were prepared using GraphPad Prism software (version 8).
